# Syntheses and crystal structures of 2,2,5-trimethyl-1,3-dioxane-5-carb­oxy­lic acid and 2,2,5-trimethyl-1,3-dioxane-5-carb­oxy­lic anhydride

**DOI:** 10.1107/S2056989019016670

**Published:** 2020-01-01

**Authors:** Joseph A. Giesen, Scott M. Grayson, Joel T. Mague

**Affiliations:** a Department of Chemistry, Tulane University, New Orleans, LA 70118, USA

**Keywords:** crystal structure, anhydride, hydrogen bond, dioxane, carb­oxy­lic acid

## Abstract

In 2,2,5-trimethyl-1,3-dioxane-5-carb­oxy­lic acid, C_8_H_14_O_4_, the carboxyl group occupies an equatorial position on the 1,3-dioxane ring. In the crystal, O—H⋯O hydrogen bonds form chains of mol­ecules, which are linked into a three-dimensional network by C—H⋯O hydrogen bonds. The asymmetric unit of 2,2,5-trimethyl-1,3-dioxane-5-carb­oxy­lic anhydride, C_16_H_26_O_7_, consists of two independent mol­ecules, which are linked by C—H⋯O hydrogen bonds. In the crystal, these units are connected into corrugated layers two mol­ecules thick and parallel to the *ab* plane by additional C—H⋯O hydrogen bonds.

## Abstract 2

The title compounds, C_8_H_14_O_4_ and C_16_H_26_O_7_, are precursors to dendrimers. The strong and weak hydrogen bonds in their extended structures are described.

## Chemical context   

Dendrimers are perfectly branched, monodisperse, multivalent polymeric structures that exhibit enhanced solubility, increased reactivity and reduced dispersity compared to linear polymer analogs (Ihre *et al.*, 1996*a* ▸). While there are several varieties of dendrimers, a protected monomer has been used to make most dendrimers (Buhleier *et al.*, 1978 ▸; Tomalia *et al.* 1985 ▸; Hawker & Fréchet, 1992 ▸). 2,2-Bis(hy­droxy­meth­yl)propionic acid (bis-MPA) is one of the most popular (Ihre *et al.*, 1996*b* ▸), useful and well-studied because of its low cost and relative ease of synthesis yielding extremely precise structures (Grayson *et al.*, 2014 ▸), while also being biocomp­atible, biodegradable and extremely modular. The synthesis of these polyester-based dendrimers relies on first protecting the hydroxyl groups of the monomer and then, after an exhaustive protection of the core, complete removal of the protecting group exposing the hydroxyl groups of the next generation. To that end, the isopropyl acetal (iso­propyl­idene/acetonide) has become one of the most commonly compounds used in the production of the monomeric unit (Stenström *et al.*, 2016 ▸; García-Gallego *et al.*, 2015 ▸). Anhydride-catalyzed esterification has become the preferred route of synthesis to produce these highly precise, bis-functional structures by decreasing the steps of purification and improving the efficiency of deprotection to the final poly-ol. The scope and diversity of these types of structures can be seen in the increase in publications on dendrimers and the numerous reviews published in recent years. We report here the syntheses and crystal structures of two important inter­mediates in our work on dendrimer syntheses, *viz*. 2,2,5-trimethyl-1,3-dioxane-5-carb­oxy­lic acid (C_8_H_14_O_4_) and 2,2,5-trimethyl-1,3-dioxane-5-carb­oxy­lic anhydride (C_16_H_26_O_7_).
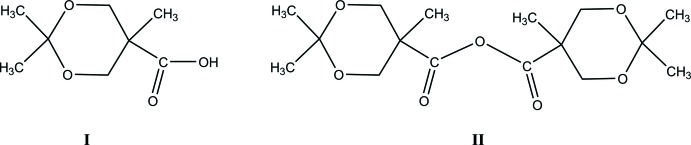


## Structural commentary   

**Figure 1:**
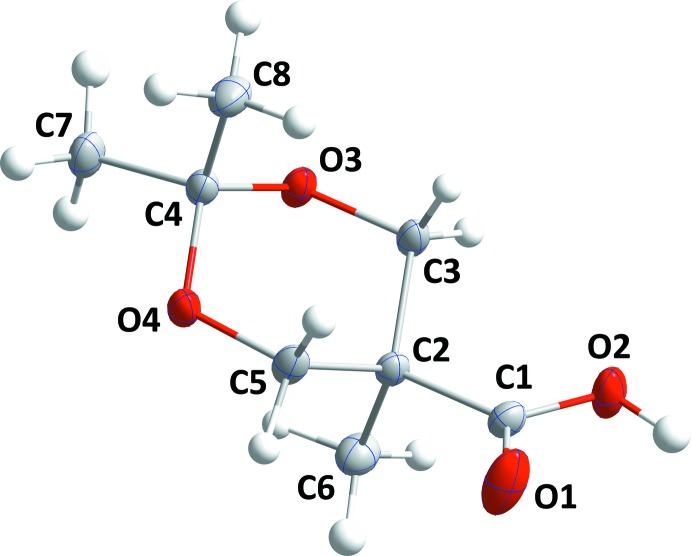
Perspective view of **I** with 50% probability displacement ellipsoids.

2,2,5-Trimethyl-1,3-dioxane-5-carb­oxy­lic acid, **I**, (Fig. 1 ▸) has the methyl groups containing C6 and C8 in *trans* axial positions while the C7 methyl group and the carboxyl group are equatorial on the 1,3-dioxane ring, which adopts an approximate chair conformation. A puckering analysis of this conformation gave the parameters *Q* = 0.5540 (9) Å, θ = 176.65 (9)° and φ = 301.8 (17)°. The O2—C1—C2—C5 torsion angle of −159.88 (8)° indicates that the carboxyl group is approximately aligned with the mean plane through the 1,3-dioxane ring.

**Figure 2:**
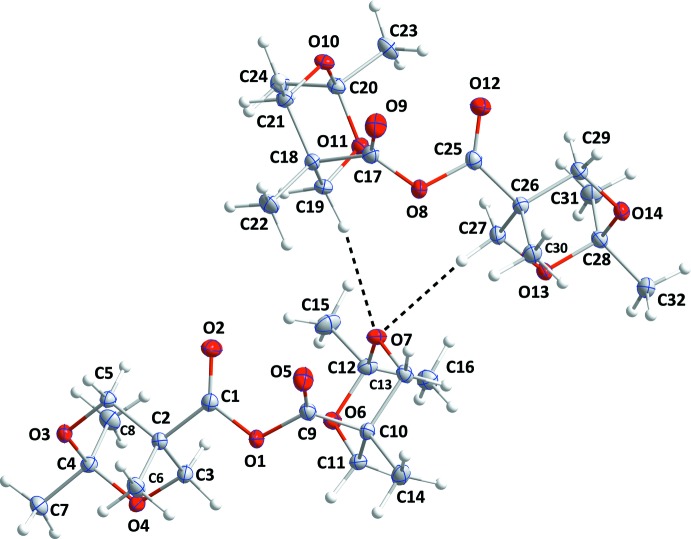
The asymmetric unit of **II** with 50% probability displacement ellipsoids. The C—H⋯O hydrogen bonds are indicated by dashed lines.

The asymmetric unit of 2,2,5-trimethyl-1,3-dioxane-5-carb­oxy­lic anhydride, **II**, consists of two independent mol­ecules each having an overall ‘U′ shape (Fig. 2 ▸) but differing in part by having opposite conformations in the anhydride portions. Thus, the O5—C9—O1—C1 and O2—C1—O1—C9 torsion angles are, respectively, 57.23 (13) and 3.46 (14)° while the O9—C17—O8—C25 and O12—C25—O8—C17 torsion angles are, respectively, −55.71 (13) and −5.51 (15)°. The positions of the substituents on the 1,3-dioxane rings are the same as for **I** and all four rings are in approximate chair forms. Puckering analyses gave *Q* = 0.5533 (10) Å, θ = 177.07 (10) and φ = 73.5 (19)° for the ring containing O3 with corresponding values of 0.5486 (10) Å, 177.14 (10) and 310 (2)°, respectively, for that containing O6, 0.5494 (10) Å, 5.32 (10) and 259.2 (11)°, respectively for that containing O10 and 0.5502 (10) Å, 4.03 (10) and 128.7 (15)°, respectively for that containing O13. In both mol­ecules, the puckering amplitudes are all comparable with the differences in the angular values resulting from the conventions used to define them (Evans & Boeyens, 1989 ▸).

## Supra­molecular features   

**Figure 3:**
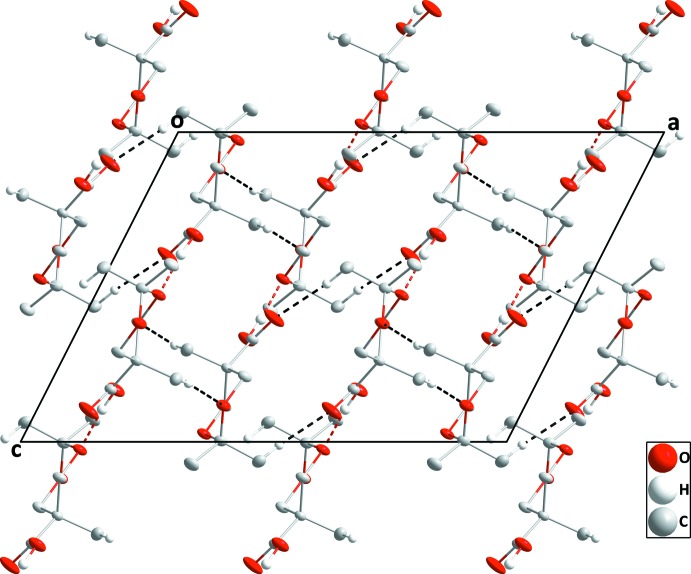
Packing of **I** viewed along the *b*-axis direction with O—H⋯O and C—H⋯O hydrogen bonds depicted, respectively, by red and black dashed lines.

**Figure 4:**
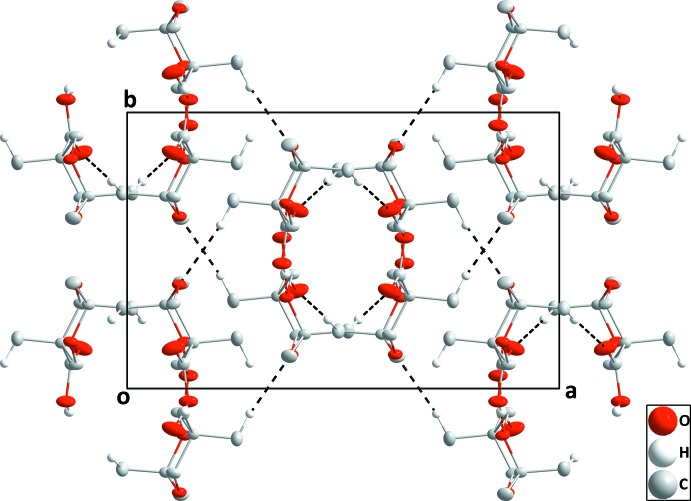
Packing of **I** viewed along the *c*-axis direction with C—H⋯O hydrogen bonds depicted by black dashed lines.

Unlike many carb­oxy­lic acids, compound **I** does not form hydrogen-bonded dimers in the crystal but rather zigzag chains along the *c*-axis direction through O2—H2⋯O3 hydrogen bonds (Table 1 ▸ and Fig. 3 ▸). These are connected into ‘tubes’ by C8—H8*B*⋯O1 hydrogen bonds (Fig. 4 ▸), with these units further linked into a three-dimensional network by C6—H6*A*⋯O4 hydrogen bonds on all sides of the ‘tube’ (Figs. 3 ▸ and 4 ▸).

**Figure 5:**
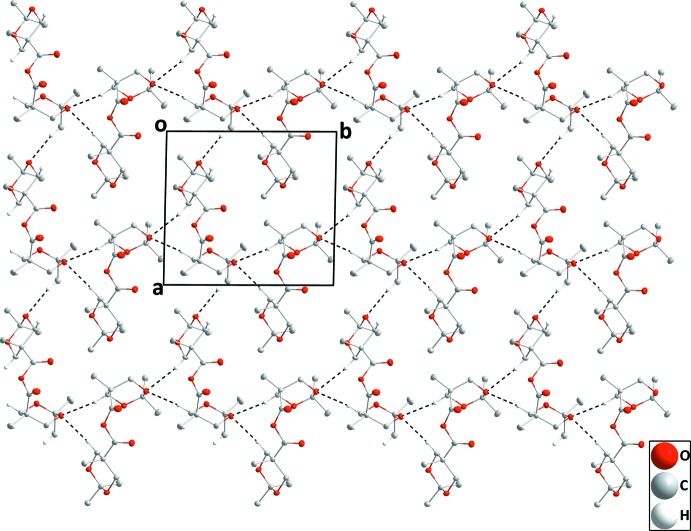
Plan view of one corrugated sheet in **II** seen along the *c*-axis direction with C—H⋯O hydrogen bonds shown as dashed lines.

**Figure 6:**
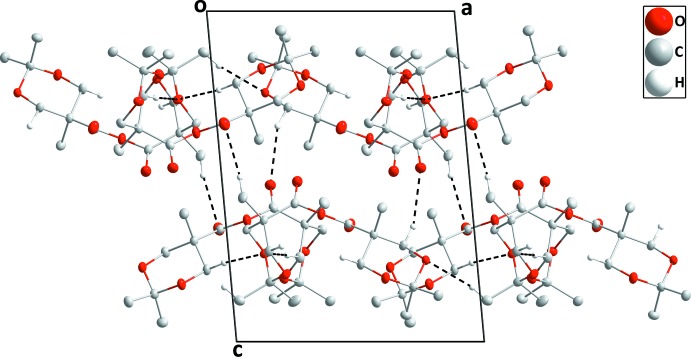
Elevation view of the double layer in **II** seen along the *b*-axis direction C—H⋯O hydrogen bonds shown as dashed lines.

The independent mol­ecules in compound **II** are connected by C19—H19*A*⋯O7 and C27—H27*A*⋯O7 hydrogen bonds (Table 2 ▸ and Fig. 5 ▸) and these units are joined into chains extending along the *b*-axis direction by C3—H3*B*⋯O10 and C11—H11*B*⋯O10 hydrogen bonds. These are linked into layers parallel to the *ab* plane by C16—H16*C*⋯O3 hydrogen bonds (Fig. 5 ▸) with two such layers joined by C5—H5*A*⋯O9 and C14—H14*A*⋯O12 hydrogen bonds (Fig. 6 ▸).

## Database survey   

A search of the Cambridge Crystallographic Database (Version 5.40, updated to September 2019; Groom *et al.*, 2016 ▸) with fragment **A** [Chem scheme2] yielded only the one structure which is closely related to **I** and **II** (**B** [Chem scheme2], WARLIN; Garmendia *et al.*, 2017 ▸). The geometry of the substituted dioxane portion here is similar to those in **I** and **II**. In the 22 additional structures found, one, **C** [Chem scheme2], (AKEKOR; Simmons *et al.*, 2011 ▸) contained a single 1,3-dioxane ring. The remaining hits were spiro­cyclic mol­ecules, *e.g*. **D** [Chem scheme2] (MINPEH; Gao *et al.*, 2018 ▸).
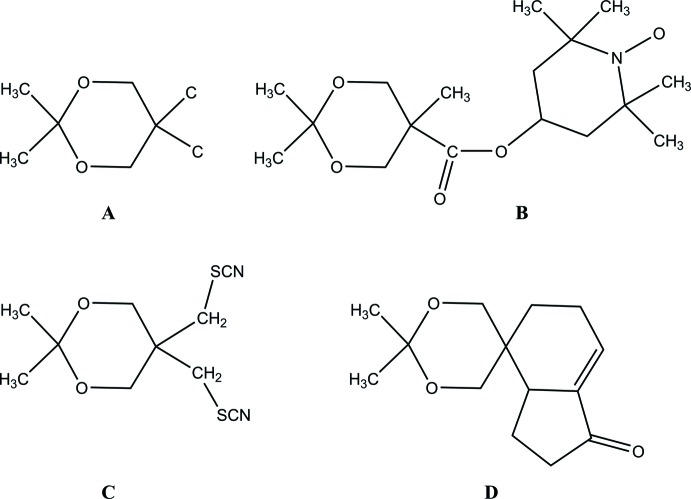


## Synthesis and crystallization   

**Preparation of 2,2,5-trimeth­oxy-1,3-dioxane-5-carb­oxy­lic acid (I):**

2,2,5-Trimeth­oxy-1,3-dioxane-5-carb­oxy­lic acid was syn­th­esized as previously reported (Ihre *et al.*, 1998 ▸; Gillies & Fréchet, 2002 ▸; Andrén *et al.*, 2017 ▸). 2,2-Bis(hy­droxy­meth­yl)propionic acid (bis-MPA, 30.68 g, 0.229 mol) was added to a 500 ml round-bottom flask equipped with a magnetic stir bar and suspended in acetone (200 ml) under stirring. 2,2-Di­meth­oxy­propane (50.0 ml, 42.5 g, 0.408 mol) and *p*-toluene­sulfonic acid monohydrate (1.17 g, 6.13 mmol) were added to the reaction flask and the residue rinsed down with acetone (50 ml). The reaction was allowed to proceed under stirring at room temperature for 8 h. Subsequently a 1:1 tri­ethyl­amine:ethanol solution (1 ml) was used to quench the reaction for 3 h. The solvent was evaporated to yield a white solid residue that was then dissolved in di­chloro­methane (DCM, 300 ml), transferred to a 500 ml separatory funnel and washed with deionized H_2_O (5 × 50 ml). The organic layer was collected in an Erlenmeyer flask equipped with a stir bar and dried over anhydrous sodium sulfate (Na_2_SO_4_) under stirring for 30 min. The Na_2_SO_4_ was removed *via* vacuum filtration, the solvent was removed by rotary evaporation, the crude product was dissolved in fresh acetone (60 ml) and recrystallized at 249 K overnight. The solid was collected by vacuum filtration *via* a fritted glass funnel and dried under high vacuum overnight to yield the protected acid as a colorless crystalline solid (17.815 g, 0.102 mol, 44.7%) ^1^H NMR (400 MHz, CDCl_3_): δ 1.20 (*s*, 3H, –CH_3_), 1.41 (*s*, 3H, –CH_3_), 1.44 (*s*, 3H, –CH_3_), 3.68 (*d*, 2H, –CH_2_O-, *J* = 12.0 Hz), 4.19 (*d*, 2H, –CH_2_O–, *J* = 12.0 Hz). ^13^C NMR (75 MHz, CDCl_3_): δ 18.48 (CH_3_), 21.89 (CH_3_), 25.59 (CH_3_), 41.82 (C), 66.11 (CH_2_), 98.55 (C), 179.52 (C).

**Synthesis of 2,2,5-trimeth­oxy-1,3-dioxane-5-carb­oxy­lic anhydride (II):**

2,2,5-Trimeth­oxy-1,3-dioxane-5-carb­oxy­lic anhydride was prepared according to the literature but with an optimized purification (Malkoch *et al.*, 2002 ▸; Giesen *et al.*, 2018 ▸). Iso­propyl­idene-protected acid (**I**, 2.334 g, 13.40 mmol) was added to a 100 ml round-bottom flask equipped with a stir bar and the solid was dissolved in di­chloro­methane (25 ml). *N*,*N*-Di­cyclo­hexyl­carbodi­imide was warmed to a liquid, transferred to a tared vial (1.349 g, 6.58 mmol) and dissolved in di­chloro­methane (10 ml). This solution was slowly added to the acid while stirring and the reaction was allowed to proceed overnight. The solid di­cyclo­hexyl­urea (DCU) that formed was removed *via* gravity filtration through fluted Q2 filter paper. The filtrate was collected and evaporated to dryness *in vacuo* affording a viscous oil that was subsequently dissolved in a minimal amount of diethyl ether under stirring and the remaining solid again removed *via* gravity filtration using Q2 filter paper. This filtrate was collected, the solvent removed, and the resulting residue dissolved in a minimal amount of warm hexa­nes. This solution was stirred overnight, affording a white solid that was removed *via* filtration and the filtrate was evaporated to yield the anhydride as a transparent viscous oil (1.956 g, 5.92 mmol, 88.4%). This was previously reported (Giesen *et al.*, 2018 ▸) and crystals of the anhydride were grown from hexa­nes. Additional purification can be achieved with removal of additional DCU by dissolving the crude viscous product in warm hexa­nes and cooling the solution at 276 K overnight to precipitate out additional DCU. This white solid was removed by vacuum filtration and the hexane evaporated yielding a transparent, viscous oil. This precipitation procedure was repeated as needed until a pure product was obtained, as judged by NMR. ^1^H NMR (400 MHz, CDCl_3_): δ 1.21 (*s*, 6H, –CH_3_), 1.42 (*s*, 6H, –CH_3_), 1.45 (*s*, 6H, –CH_3_), 3.68 (*d*, 4H, –CH_2_O–, *J* = 12.0 Hz), 4.18 (*d*, 4H, –CH_2_O–, *J* = 12.0 Hz). ^13^C NMR (100 MHz, CDCl_3_): δ 17.80 (CH_3_), 21.70 (CH_3_), 25.70 (CH_3_), 43.79 (C), 65.81 (CH2), 98.53 (C), 169.63 (C). Elemental analysis: calculated for C_16_H_26_O_7_: C, 58.17; H, 7.93; 33.90. Found: C, 57.29; H, 8.30; O, 34.22.

## Refinement   

Crystal data, data collection and structure refinement details are summarized in Table 3 ▸. H atoms in **II** were included as riding contributions in idealized positions with C—H = 0.98–0.99 Å and *U*_iso_(H) = 1.2*U*_eq_(C) or 1.5*U*_eq_(C-methyl).

## Supplementary Material

Crystal structure: contains datablock(s) global, I, II. DOI: 10.1107/S2056989019016670/hb7867sup1.cif

Structure factors: contains datablock(s) I. DOI: 10.1107/S2056989019016670/hb7867Isup2.hkl

Structure factors: contains datablock(s) II. DOI: 10.1107/S2056989019016670/hb7867IIsup3.hkl

13C and 1H NMR spectra. DOI: 10.1107/S2056989019016670/hb7867sup4.docx

CCDC references: 1971440, 1971439

Additional supporting information:  crystallographic information; 3D view; checkCIF report

## Figures and Tables

**Table 1:** Hydrogen-bond geometry (Å, °) for **I** [Chem scheme1]

*D*—H⋯*A*	*D*—H	H⋯*A*	*D*⋯*A*	*D*—H⋯*A*
O2—H2⋯O3^i^	0.909 (17)	1.804 (17)	2.7086 (9)	172.6 (14)
C6—H6*A*⋯O4^ii^	0.979 (15)	2.527 (15)	3.4958 (13)	170.4 (12)
C8—H8*B*⋯O1^iii^	0.984 (14)	2.405 (14)	3.3864 (12)	174.8 (11)

Symmetry codes: (i) 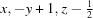; (ii) 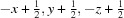; (iii) 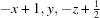.

**Table 2:** Hydrogen-bond geometry (Å, °) for (II)

*D*—H⋯*A*	*D*—H	H⋯*A*	*D*⋯*A*	*D*—H⋯*A*
C3—H3*B*⋯O10^i^	0.99	2.54	3.5043 (16)	164
C5—H5*A*⋯O9^ii^	0.99	2.54	3.4723 (18)	156
C11—H11*B*⋯O10^i^	0.99	2.57	3.5152 (17)	161
C14—H14*A*⋯O12^iii^	0.98	2.56	3.531 (2)	171
C16—H16*C*⋯O3^iv^	0.98	2.53	3.4973 (16)	170
C19—H19*A*⋯O7	0.99	2.53	3.5095 (17)	168
C27—H27*A*⋯O7	0.99	2.52	3.5035 (16)	170

Symmetry codes: (i) 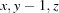; (ii) 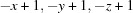; (iii) 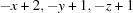; (iv) 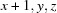.

**Table 3:** Experimental details

	**I**	**II**
Crystal data		
Chemical formula	C_8_H_14_O_4_	C_16_H_26_O_7_
*M*_r_	174.19	330.37
Crystal system, space group	Monoclinic, *C*2/*c*	Triclinic, *P* 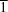
Temperature (K)	150	100
*a*, *b*, *c* (Å)	16.9457 (8), 9.6453 (5), 12.1052 (6)	10.355 (4), 11.928 (5), 14.496 (6)
α, β, γ (°)	90, 116.986 (1), 90	73.128 (5), 84.900 (5), 89.499 (6)
*V* (Å^3^)	1763.12 (15)	1706.3 (11)
*Z*	8	4
Radiation type	Mo *K*α	Mo *K*α
μ (mm^−1^)	0.11	0.10
Crystal size (mm)	0.35 × 0.32 × 0.25	0.30 × 0.30 × 0.22

Data collection		
Diffractometer	Bruker SMART APEX CCD	Bruker SMART APEX CCD
Absorption correction	Multi-scan (*SADABS*; Krause *et al.*, 2015 ▸)	Multi-scan (*SADABS*; Krause *et al.*, 2015 ▸)
*T*_min_, *T*_max_	0.91, 0.97	0.97, 0.98
No. of measured, independent and observed [*I* > 2σ(*I*)] reflections	16506, 2367, 2035	30041, 8606, 7451
*R*_int_	0.026	0.044
(sin θ/λ)_max_ (Å^−1^)	0.685	0.687

Refinement		
*R*[*F* ^2^ > 2σ(*F* ^2^)], *wR*(*F* ^2^), *S*	0.039, 0.113, 1.07	0.040, 0.109, 1.04
No. of reflections	2367	8606
No. of parameters	165	427
H-atom treatment	All H-atom parameters refined	H-atom parameters constrained
Δρ_max_, Δρ_min_ (e Å^−3^)	0.44, −0.18	0.35, −0.32

Computer programs: *APEX3* and *SAINT* (Bruker, 2016 ▸), *SHELXT* (Sheldrick, 2015*a* ▸), *SHELXL2018* (Sheldrick, 2015*b* ▸), *DIAMOND* (Brandenburg & Putz, 2012 ▸) and *SHELXTL* (Sheldrick, 2008 ▸).

## supplementary crystallographic information

### 2,2,5-Trimethyl-1,3-dioxane-5-carboxylic acid (I) Crystal data

**Table:**

C_8_H_14_O_4_	*F*(000) = 752
*M**_r_* = 174.19	*D*_x_ = 1.312 Mg m^−^^3^
Monoclinic, *C*2/*c*	Mo *K*α radiation, λ = 0.71073 Å
*a* = 16.9457 (8) Å	Cell parameters from 8540 reflections
*b* = 9.6453 (5) Å	θ = 2.5–29.1°
*c* = 12.1052 (6) Å	µ = 0.11 mm^−^^1^
β = 116.986 (1)°	*T* = 150 K
*V* = 1763.12 (15) Å^3^	Block, colourless
*Z* = 8	0.35 × 0.32 × 0.25 mm

### 2,2,5-Trimethyl-1,3-dioxane-5-carboxylic acid (I) Data collection

**Table:**

Bruker SMART APEX CCD diffractometer	2367 independent reflections
Radiation source: fine-focus sealed tube	2035 reflections with *I* > 2σ(*I*)
Graphite monochromator	*R*_int_ = 0.026
Detector resolution: 8.3333 pixels mm^-1^	θ_max_ = 29.1°, θ_min_ = 2.5°
φ and ω scans	*h* = −23→23
Absorption correction: multi-scan (*SADABS*; Krause *et al.*, 2015)	*k* = −13→13
*T*_min_ = 0.91, *T*_max_ = 0.97	*l* = −16→16
16506 measured reflections	

### 2,2,5-Trimethyl-1,3-dioxane-5-carboxylic acid (I) Refinement

**Table:**

Refinement on *F*^2^	Primary atom site location: dual
Least-squares matrix: full	Secondary atom site location: difference Fourier map
*R*[*F*^2^ > 2σ(*F*^2^)] = 0.039	Hydrogen site location: difference Fourier map
*wR*(*F*^2^) = 0.113	All H-atom parameters refined
*S* = 1.07	*w* = 1/[σ^2^(*F*_o_^2^) + (0.0774*P*)^2^ + 0.3458*P*] where *P* = (*F*_o_^2^ + 2*F*_c_^2^)/3
2367 reflections	(Δ/σ)_max_ = 0.001
165 parameters	Δρ_max_ = 0.44 e Å^−^^3^
0 restraints	Δρ_min_ = −0.18 e Å^−^^3^

### 2,2,5-Trimethyl-1,3-dioxane-5-carboxylic acid (I) Special details

**Table:**

Experimental. The diffraction data were obtained from 3 sets of 400 frames, each of width 0.5° in ω, colllected at φ = 0.00, 90.00 and 180.00° and 2 sets of 800 frames, each of width 0.45° in φ, collected at ω = –30.00 and 210.00°. The scan time was 10 sec/frame.
Geometry. All esds (except the esd in the dihedral angle between two l.s. planes) are estimated using the full covariance matrix. The cell esds are taken into account individually in the estimation of esds in distances, angles and torsion angles; correlations between esds in cell parameters are only used when they are defined by crystal symmetry. An approximate (isotropic) treatment of cell esds is used for estimating esds involving l.s. planes.
Refinement. Refinement of F^2^ against ALL reflections. The weighted R-factor wR and goodness of fit S are based on F^2^, conventional R-factors R are based on F, with F set to zero for negative F^2^. The threshold expression of F^2^ > 2sigma(F^2^) is used only for calculating R-factors(gt) etc. and is not relevant to the choice of reflections for refinement. R-factors based on F^2^ are statistically about twice as large as those based on F, and R- factors based on ALL data will be even larger.

### 2,2,5-Trimethyl-1,3-dioxane-5-carboxylic acid (I) Fractional atomic coordinates and isotropic or equivalent isotropic displacement parameters (Å^2^)

**Table:**

	*x*	*y*	*z*	*U*_iso_*/*U*_eq_	
O1	0.39277 (7)	0.35303 (8)	0.10104 (8)	0.0395 (2)	
O2	0.35826 (5)	0.54688 (7)	0.16844 (7)	0.0280 (2)	
H2	0.3661 (10)	0.5831 (16)	0.1048 (16)	0.044 (4)*	
O3	0.36941 (4)	0.33124 (7)	0.47442 (6)	0.01898 (17)	
O4	0.37935 (4)	0.12345 (7)	0.38268 (6)	0.01931 (17)	
C1	0.36963 (6)	0.41082 (10)	0.16968 (8)	0.01841 (19)	
C2	0.34712 (6)	0.33493 (9)	0.26155 (7)	0.01538 (18)	
C3	0.38203 (6)	0.41384 (9)	0.38447 (8)	0.01846 (19)	
H3	0.4442 (8)	0.4359 (13)	0.4139 (12)	0.025 (3)*	
H3B	0.3511 (9)	0.5007 (14)	0.3792 (12)	0.028 (3)*	
C4	0.41050 (6)	0.19614 (9)	0.49632 (8)	0.0176 (2)	
C5	0.39283 (6)	0.19347 (9)	0.28855 (8)	0.0194 (2)	
H5	0.4560 (9)	0.2045 (15)	0.3127 (13)	0.034 (3)*	
H5B	0.3649 (9)	0.1329 (14)	0.2151 (13)	0.031 (3)*	
C6	0.24588 (6)	0.31871 (11)	0.20277 (9)	0.0251 (2)	
H6A	0.2160 (9)	0.4086 (15)	0.1885 (13)	0.031 (3)*	
H6B	0.2316 (8)	0.2611 (14)	0.2616 (12)	0.028 (3)*	
H6C	0.2246 (10)	0.2711 (16)	0.1240 (14)	0.042 (4)*	
C7	0.37499 (7)	0.11697 (11)	0.57215 (9)	0.0252 (2)	
H7A	0.3976 (10)	0.1590 (16)	0.6557 (15)	0.045 (4)*	
H7B	0.3967 (10)	0.0208 (16)	0.5805 (14)	0.037 (4)*	
H7C	0.3113 (10)	0.1106 (16)	0.5286 (14)	0.039 (4)*	
C8	0.51093 (6)	0.20861 (11)	0.56270 (9)	0.0256 (2)	
H8A	0.5272 (10)	0.2602 (16)	0.6373 (14)	0.039 (4)*	
H8B	0.5353 (9)	0.2497 (15)	0.5103 (13)	0.034 (3)*	
H8C	0.5394 (9)	0.1170 (16)	0.5863 (13)	0.034 (3)*	

### 2,2,5-Trimethyl-1,3-dioxane-5-carboxylic acid (I) Atomic displacement parameters (Å^2^)

**Table:**

	*U*^11^	*U*^22^	*U*^33^	*U*^12^	*U*^13^	*U*^23^
O1	0.0713 (6)	0.0306 (4)	0.0381 (5)	0.0155 (4)	0.0436 (5)	0.0086 (3)
O2	0.0493 (5)	0.0185 (3)	0.0265 (4)	0.0005 (3)	0.0263 (3)	0.0028 (3)
O3	0.0299 (3)	0.0157 (3)	0.0163 (3)	0.0032 (2)	0.0147 (3)	0.0010 (2)
O4	0.0287 (3)	0.0139 (3)	0.0173 (3)	−0.0008 (2)	0.0122 (3)	−0.0004 (2)
C1	0.0209 (4)	0.0207 (4)	0.0148 (4)	0.0014 (3)	0.0091 (3)	0.0013 (3)
C2	0.0189 (4)	0.0159 (4)	0.0134 (4)	0.0005 (3)	0.0091 (3)	0.0000 (3)
C3	0.0285 (4)	0.0144 (4)	0.0152 (4)	−0.0004 (3)	0.0123 (3)	−0.0001 (3)
C4	0.0229 (4)	0.0149 (4)	0.0158 (4)	0.0005 (3)	0.0094 (3)	0.0013 (3)
C5	0.0284 (4)	0.0163 (4)	0.0172 (4)	0.0037 (3)	0.0136 (3)	0.0003 (3)
C6	0.0195 (4)	0.0307 (5)	0.0232 (5)	−0.0006 (4)	0.0081 (4)	0.0039 (4)
C7	0.0348 (5)	0.0233 (5)	0.0227 (5)	−0.0022 (4)	0.0175 (4)	0.0037 (4)
C8	0.0219 (4)	0.0301 (5)	0.0207 (5)	−0.0002 (4)	0.0062 (4)	0.0055 (4)

### 2,2,5-Trimethyl-1,3-dioxane-5-carboxylic acid (I) Geometric parameters (Å, º)

**Table:**

O1—C1	1.2043 (11)	C4—C7	1.5129 (12)
O2—C1	1.3255 (12)	C4—C8	1.5216 (12)
O2—H2	0.909 (17)	C5—H5	0.979 (14)
O3—C3	1.4414 (10)	C5—H5B	0.986 (14)
O3—C4	1.4442 (11)	C6—H6A	0.979 (14)
O4—C4	1.4155 (10)	C6—H6B	1.015 (13)
O4—C5	1.4294 (10)	C6—H6C	0.968 (15)
C1—C2	1.5176 (11)	C7—H7A	0.991 (16)
C2—C5	1.5293 (12)	C7—H7B	0.986 (15)
C2—C3	1.5312 (12)	C7—H7C	0.964 (15)
C2—C6	1.5382 (12)	C8—H8A	0.955 (15)
C3—H3	0.970 (13)	C8—H8B	0.984 (14)
C3—H3B	0.975 (13)	C8—H8C	0.985 (15)
			
C1—O2—H2	108.3 (10)	O4—C5—C2	110.07 (7)
C3—O3—C4	114.39 (6)	O4—C5—H5	111.2 (8)
C4—O4—C5	114.63 (7)	C2—C5—H5	110.2 (9)
O1—C1—O2	122.79 (8)	O4—C5—H5B	104.7 (8)
O1—C1—C2	123.44 (9)	C2—C5—H5B	110.3 (8)
O2—C1—C2	113.72 (7)	H5—C5—H5B	110.2 (11)
C1—C2—C5	108.39 (7)	C2—C6—H6A	111.8 (8)
C1—C2—C3	110.95 (7)	C2—C6—H6B	107.6 (7)
C5—C2—C3	107.51 (7)	H6A—C6—H6B	109.9 (11)
C1—C2—C6	107.98 (7)	C2—C6—H6C	110.0 (9)
C5—C2—C6	111.02 (8)	H6A—C6—H6C	108.3 (13)
C3—C2—C6	110.98 (7)	H6B—C6—H6C	109.3 (12)
O3—C3—C2	109.57 (7)	C4—C7—H7A	109.6 (9)
O3—C3—H3	110.6 (8)	C4—C7—H7B	107.7 (8)
C2—C3—H3	110.0 (7)	H7A—C7—H7B	109.1 (13)
O3—C3—H3B	105.3 (8)	C4—C7—H7C	110.8 (9)
C2—C3—H3B	113.8 (8)	H7A—C7—H7C	113.7 (13)
H3—C3—H3B	107.3 (11)	H7B—C7—H7C	105.7 (13)
O4—C4—O3	109.42 (7)	C4—C8—H8A	108.3 (9)
O4—C4—C7	105.31 (7)	C4—C8—H8B	112.8 (8)
O3—C4—C7	105.98 (7)	H8A—C8—H8B	112.0 (13)
O4—C4—C8	112.76 (7)	C4—C8—H8C	111.4 (8)
O3—C4—C8	110.86 (7)	H8A—C8—H8C	107.4 (13)
C7—C4—C8	112.14 (8)	H8B—C8—H8C	104.8 (12)
			
O1—C1—C2—C5	22.55 (12)	C5—O4—C4—O3	56.51 (9)
O2—C1—C2—C5	−159.88 (8)	C5—O4—C4—C7	170.04 (7)
O1—C1—C2—C3	140.38 (10)	C5—O4—C4—C8	−67.36 (10)
O2—C1—C2—C3	−42.05 (10)	C3—O3—C4—O4	−56.03 (9)
O1—C1—C2—C6	−97.79 (11)	C3—O3—C4—C7	−169.12 (7)
O2—C1—C2—C6	79.77 (10)	C3—O3—C4—C8	68.95 (9)
C4—O3—C3—C2	57.03 (9)	C4—O4—C5—C2	−58.36 (10)
C1—C2—C3—O3	−172.93 (7)	C1—C2—C5—O4	175.13 (7)
C5—C2—C3—O3	−54.57 (9)	C3—C2—C5—O4	55.13 (9)
C6—C2—C3—O3	67.02 (9)	C6—C2—C5—O4	−66.43 (9)

### 2,2,5-Trimethyl-1,3-dioxane-5-carboxylic acid (I) Hydrogen-bond geometry (Å, º)

**Table:**

*D*—H···*A*	*D*—H	H···*A*	*D*···*A*	*D*—H···*A*
O2—H2···O3^i^	0.909 (17)	1.804 (17)	2.7086 (9)	172.6 (14)
C6—H6*A*···O4^ii^	0.979 (15)	2.527 (15)	3.4958 (13)	170.4 (12)
C8—H8*B*···O1^iii^	0.984 (14)	2.405 (14)	3.3864 (12)	174.8 (11)

Symmetry codes: (i) *x*, −*y*+1, *z*−1/2; (ii) −*x*+1/2, *y*+1/2, −*z*+1/2; (iii) −*x*+1, *y*, −*z*+1/2.

### 2,2,5-Trimethyl-1,3-dioxane-5-carboxylic anhydride (II) Crystal data

**Table:**

C_16_H_26_O_7_	*Z* = 4
*M**_r_* = 330.37	*F*(000) = 712
Triclinic, *P*1	*D*_x_ = 1.286 Mg m^−^^3^
*a* = 10.355 (4) Å	Mo *K*α radiation, λ = 0.71073 Å
*b* = 11.928 (5) Å	Cell parameters from 9690 reflections
*c* = 14.496 (6) Å	θ = 2.4–29.6°
α = 73.128 (5)°	µ = 0.10 mm^−^^1^
β = 84.900 (5)°	*T* = 100 K
γ = 89.499 (6)°	Block, colourless
*V* = 1706.3 (11) Å^3^	0.30 × 0.30 × 0.22 mm

### 2,2,5-Trimethyl-1,3-dioxane-5-carboxylic anhydride (II) Data collection

**Table:**

Bruker SMART APEX CCD diffractometer	8606 independent reflections
Radiation source: fine-focus sealed tube	7451 reflections with *I* > 2σ(*I*)
Graphite monochromator	*R*_int_ = 0.044
Detector resolution: 8.3333 pixels mm^-1^	θ_max_ = 29.3°, θ_min_ = 2.0°
φ and ω scans	*h* = −13→14
Absorption correction: multi-scan (*SADABS*; Krause *et al.*, 2015)	*k* = −16→15
*T*_min_ = 0.97, *T*_max_ = 0.98	*l* = −19→19
30041 measured reflections	

### 2,2,5-Trimethyl-1,3-dioxane-5-carboxylic anhydride (II) Refinement

**Table:**

Refinement on *F*^2^	Primary atom site location: dual
Least-squares matrix: full	Secondary atom site location: difference Fourier map
*R*[*F*^2^ > 2σ(*F*^2^)] = 0.040	Hydrogen site location: inferred from neighbouring sites
*wR*(*F*^2^) = 0.109	H-atom parameters constrained
*S* = 1.04	*w* = 1/[σ^2^(*F*_o_^2^) + (0.0556*P*)^2^ + 0.3868*P*] where *P* = (*F*_o_^2^ + 2*F*_c_^2^)/3
8606 reflections	(Δ/σ)_max_ = 0.001
427 parameters	Δρ_max_ = 0.35 e Å^−^^3^
0 restraints	Δρ_min_ = −0.32 e Å^−^^3^

### 2,2,5-Trimethyl-1,3-dioxane-5-carboxylic anhydride (II) Special details

**Table:**

Experimental. The diffraction data were obtained from 3 sets of 400 frames, each of width 0.5° in ω, colllected at φ = 0.00, 90.00 and 180.00° and 2 sets of 800 frames, each of width 0.45° in φ, collected at ω = –30.00 and 210.00°. The scan time was 20 sec/frame.
Geometry. All esds (except the esd in the dihedral angle between two l.s. planes) are estimated using the full covariance matrix. The cell esds are taken into account individually in the estimation of esds in distances, angles and torsion angles; correlations between esds in cell parameters are only used when they are defined by crystal symmetry. An approximate (isotropic) treatment of cell esds is used for estimating esds involving l.s. planes.
Refinement. Refinement of F^2^ against ALL reflections. The weighted R-factor wR and goodness of fit S are based on F^2^, conventional R-factors R are based on F, with F set to zero for negative F^2^. The threshold expression of F^2^ > 2sigma(F^2^) is used only for calculating R-factors(gt) etc. and is not relevant to the choice of reflections for refinement. R-factors based on F^2^ are statistically about twice as large as those based on F, and R- factors based on ALL data will be even larger. H-atoms attached to carbon were placed in calculated positions (C—H = 0.95 - 0.98 Å). All were included as riding contributions with isotropic displacement parameters 1.2 - 1.5 times those of the attached atoms.

### 2,2,5-Trimethyl-1,3-dioxane-5-carboxylic anhydride (II) Fractional atomic coordinates and isotropic or equivalent isotropic displacement parameters (Å^2^)

**Table:**

	*x*	*y*	*z*	*U*_iso_*/*U*_eq_	
O1	0.61114 (7)	0.17397 (6)	0.38614 (5)	0.01811 (15)	
O2	0.50096 (8)	0.34387 (7)	0.35198 (6)	0.02453 (17)	
O3	0.20648 (6)	0.19286 (6)	0.24679 (5)	0.01536 (14)	
O4	0.37528 (7)	0.08851 (6)	0.19282 (5)	0.01796 (15)	
O5	0.69640 (8)	0.26493 (7)	0.48443 (6)	0.02468 (17)	
O6	0.77584 (7)	0.26736 (6)	0.20400 (5)	0.01762 (15)	
O7	0.85266 (7)	0.41175 (6)	0.26717 (5)	0.01698 (15)	
C1	0.50623 (9)	0.24255 (9)	0.35590 (7)	0.01594 (19)	
C2	0.40734 (9)	0.17221 (8)	0.32402 (7)	0.01479 (19)	
C3	0.47061 (9)	0.13905 (9)	0.23488 (7)	0.01764 (19)	
H3A	0.509546	0.209880	0.186491	0.021*	
H3B	0.540504	0.082334	0.254818	0.021*	
C4	0.26705 (9)	0.16139 (9)	0.16544 (7)	0.01538 (19)	
C5	0.29198 (9)	0.25081 (9)	0.29146 (7)	0.01568 (19)	
H5A	0.244737	0.268096	0.348059	0.019*	
H5B	0.323569	0.325907	0.244884	0.019*	
C6	0.36290 (10)	0.06178 (9)	0.40559 (8)	0.0201 (2)	
H6A	0.437550	0.011721	0.423812	0.030*	
H6B	0.298009	0.018822	0.383227	0.030*	
H6C	0.324777	0.084269	0.461837	0.030*	
C7	0.16859 (10)	0.08444 (9)	0.14008 (8)	0.0197 (2)	
H7A	0.137648	0.022159	0.198378	0.030*	
H7B	0.209200	0.049489	0.091533	0.030*	
H7C	0.095236	0.131909	0.113908	0.030*	
C8	0.30637 (11)	0.26922 (9)	0.08043 (7)	0.0213 (2)	
H8A	0.231143	0.319476	0.065223	0.032*	
H8B	0.337824	0.244353	0.023790	0.032*	
H8C	0.375355	0.312944	0.097812	0.032*	
C9	0.71419 (10)	0.22328 (9)	0.41881 (7)	0.01674 (19)	
C10	0.84583 (9)	0.20922 (8)	0.36723 (7)	0.01496 (18)	
C11	0.83536 (10)	0.17628 (8)	0.27399 (7)	0.01655 (19)	
H11A	0.923038	0.161976	0.246924	0.020*	
H11B	0.783369	0.102966	0.288491	0.020*	
C12	0.84132 (10)	0.37818 (9)	0.18094 (7)	0.01700 (19)	
C13	0.91677 (10)	0.32801 (8)	0.33964 (7)	0.01650 (19)	
H13A	0.918581	0.355697	0.397628	0.020*	
H13B	1.007414	0.319629	0.314757	0.020*	
C14	0.92243 (10)	0.11550 (9)	0.43651 (8)	0.0221 (2)	
H14A	0.925743	0.135002	0.497449	0.033*	
H14B	1.010801	0.112858	0.406871	0.033*	
H14C	0.879586	0.038874	0.449389	0.033*	
C15	0.75121 (12)	0.46587 (10)	0.12292 (9)	0.0268 (2)	
H15A	0.667202	0.462130	0.160693	0.040*	
H15B	0.739079	0.447442	0.062590	0.040*	
H15C	0.788927	0.544884	0.107845	0.040*	
C16	0.97334 (11)	0.37799 (9)	0.12476 (8)	0.0216 (2)	
H16A	1.013381	0.456339	0.107417	0.032*	
H16B	0.961933	0.356256	0.065737	0.032*	
H16C	1.029383	0.321247	0.165093	0.032*	
O8	0.91353 (7)	0.66447 (6)	0.36711 (5)	0.01908 (16)	
O9	0.80649 (8)	0.74653 (7)	0.47388 (6)	0.02506 (17)	
O10	0.69272 (7)	0.91031 (6)	0.27307 (5)	0.01648 (15)	
O11	0.78656 (7)	0.78188 (6)	0.19087 (5)	0.01714 (15)	
O12	1.03035 (8)	0.83256 (7)	0.34106 (6)	0.02418 (17)	
O13	1.20111 (7)	0.58226 (6)	0.17388 (5)	0.01848 (15)	
O14	1.35295 (7)	0.69110 (6)	0.22514 (5)	0.01777 (15)	
C17	0.80283 (10)	0.71100 (9)	0.40517 (7)	0.01728 (19)	
C18	0.68200 (9)	0.70215 (8)	0.35467 (7)	0.01530 (19)	
C19	0.71338 (10)	0.68437 (8)	0.25503 (7)	0.01675 (19)	
H19A	0.763701	0.611930	0.261691	0.020*	
H19B	0.631671	0.674776	0.227257	0.020*	
C20	0.72594 (10)	0.89232 (9)	0.18016 (7)	0.01614 (19)	
C21	0.61304 (10)	0.81907 (9)	0.33904 (7)	0.01696 (19)	
H21A	0.529030	0.814447	0.312520	0.020*	
H21B	0.595578	0.836590	0.401641	0.020*	
C22	0.59509 (11)	0.60097 (9)	0.41984 (8)	0.0232 (2)	
H22A	0.514044	0.598390	0.390309	0.035*	
H22B	0.575684	0.613336	0.483547	0.035*	
H22C	0.640143	0.526714	0.427203	0.035*	
C23	0.83068 (11)	0.98287 (10)	0.13041 (8)	0.0226 (2)	
H23A	0.903529	0.973284	0.170656	0.034*	
H23B	0.795553	1.061567	0.120902	0.034*	
H23C	0.860766	0.972364	0.067464	0.034*	
C24	0.60689 (10)	0.90425 (9)	0.12294 (8)	0.0196 (2)	
H24A	0.633618	0.901436	0.057087	0.029*	
H24B	0.565364	0.979148	0.120183	0.029*	
H24C	0.545396	0.839795	0.154879	0.029*	
C25	1.02532 (10)	0.73291 (9)	0.34016 (7)	0.01695 (19)	
C26	1.13437 (9)	0.66492 (8)	0.30582 (7)	0.01591 (19)	
C27	1.09366 (9)	0.63078 (9)	0.21756 (7)	0.0179 (2)	
H27A	1.021396	0.572587	0.238381	0.022*	
H27B	1.062981	0.700902	0.169665	0.022*	
C28	1.31372 (10)	0.65704 (9)	0.14491 (7)	0.0171 (2)	
C29	1.25393 (10)	0.74666 (9)	0.27107 (8)	0.0182 (2)	
H29A	1.229428	0.820862	0.224740	0.022*	
H29B	1.287269	0.765368	0.326907	0.022*	
C30	1.16462 (10)	0.55598 (9)	0.38751 (8)	0.0207 (2)	
H30A	1.190672	0.580001	0.442426	0.031*	
H30B	1.235311	0.513214	0.364143	0.031*	
H30C	1.087204	0.505140	0.408148	0.031*	
C31	1.29214 (11)	0.76289 (10)	0.05887 (8)	0.0236 (2)	
H31A	1.221283	0.809470	0.077099	0.035*	
H31B	1.269615	0.736065	0.004421	0.035*	
H31C	1.371653	0.811087	0.039866	0.035*	
C32	1.42178 (10)	0.58090 (9)	0.12077 (8)	0.0211 (2)	
H32A	1.501898	0.627907	0.099325	0.032*	
H32B	1.398129	0.549594	0.068966	0.032*	
H32C	1.435184	0.515969	0.178409	0.032*	

### 2,2,5-Trimethyl-1,3-dioxane-5-carboxylic anhydride (II) Atomic displacement parameters (Å^2^)

**Table:**

	*U*^11^	*U*^22^	*U*^33^	*U*^12^	*U*^13^	*U*^23^
O1	0.0127 (3)	0.0173 (3)	0.0257 (4)	0.0006 (3)	−0.0049 (3)	−0.0074 (3)
O2	0.0246 (4)	0.0174 (4)	0.0347 (4)	0.0021 (3)	−0.0099 (3)	−0.0105 (3)
O3	0.0121 (3)	0.0186 (3)	0.0166 (3)	0.0000 (3)	−0.0012 (2)	−0.0071 (3)
O4	0.0146 (3)	0.0178 (3)	0.0248 (4)	0.0030 (3)	−0.0044 (3)	−0.0107 (3)
O5	0.0211 (4)	0.0332 (4)	0.0234 (4)	0.0008 (3)	−0.0028 (3)	−0.0138 (3)
O6	0.0183 (3)	0.0160 (3)	0.0203 (3)	0.0012 (3)	−0.0070 (3)	−0.0066 (3)
O7	0.0213 (4)	0.0127 (3)	0.0178 (3)	0.0020 (3)	−0.0047 (3)	−0.0049 (3)
C1	0.0139 (4)	0.0172 (5)	0.0162 (4)	0.0006 (4)	−0.0013 (3)	−0.0041 (4)
C2	0.0124 (4)	0.0144 (4)	0.0178 (4)	0.0000 (3)	−0.0021 (3)	−0.0049 (3)
C3	0.0124 (4)	0.0200 (5)	0.0231 (5)	0.0012 (4)	−0.0020 (4)	−0.0103 (4)
C4	0.0140 (4)	0.0162 (4)	0.0166 (4)	0.0011 (3)	−0.0008 (3)	−0.0060 (4)
C5	0.0141 (4)	0.0160 (4)	0.0186 (4)	0.0017 (3)	−0.0034 (3)	−0.0073 (4)
C6	0.0196 (5)	0.0176 (5)	0.0207 (5)	−0.0032 (4)	−0.0043 (4)	−0.0011 (4)
C7	0.0183 (5)	0.0199 (5)	0.0226 (5)	−0.0022 (4)	−0.0039 (4)	−0.0082 (4)
C8	0.0240 (5)	0.0211 (5)	0.0172 (5)	−0.0030 (4)	0.0005 (4)	−0.0034 (4)
C9	0.0146 (4)	0.0161 (4)	0.0189 (5)	−0.0009 (4)	−0.0035 (3)	−0.0036 (4)
C10	0.0127 (4)	0.0143 (4)	0.0177 (4)	0.0005 (3)	−0.0037 (3)	−0.0037 (3)
C11	0.0168 (4)	0.0131 (4)	0.0204 (5)	0.0015 (3)	−0.0031 (4)	−0.0057 (4)
C12	0.0200 (5)	0.0147 (4)	0.0168 (4)	0.0030 (4)	−0.0042 (4)	−0.0047 (4)
C13	0.0158 (4)	0.0154 (4)	0.0179 (4)	−0.0013 (4)	−0.0054 (4)	−0.0032 (4)
C14	0.0179 (5)	0.0202 (5)	0.0244 (5)	0.0031 (4)	−0.0052 (4)	0.0005 (4)
C15	0.0323 (6)	0.0230 (5)	0.0254 (5)	0.0106 (5)	−0.0115 (5)	−0.0051 (4)
C16	0.0236 (5)	0.0200 (5)	0.0195 (5)	0.0001 (4)	0.0007 (4)	−0.0038 (4)
O8	0.0130 (3)	0.0183 (3)	0.0265 (4)	−0.0002 (3)	−0.0001 (3)	−0.0078 (3)
O9	0.0234 (4)	0.0331 (4)	0.0206 (4)	0.0007 (3)	−0.0015 (3)	−0.0110 (3)
O10	0.0210 (4)	0.0126 (3)	0.0167 (3)	−0.0014 (3)	−0.0006 (3)	−0.0059 (3)
O11	0.0163 (3)	0.0159 (3)	0.0197 (3)	−0.0001 (3)	0.0016 (3)	−0.0068 (3)
O12	0.0233 (4)	0.0181 (4)	0.0319 (4)	−0.0012 (3)	0.0012 (3)	−0.0095 (3)
O13	0.0138 (3)	0.0179 (3)	0.0250 (4)	−0.0027 (3)	0.0002 (3)	−0.0088 (3)
O14	0.0134 (3)	0.0195 (4)	0.0215 (4)	−0.0015 (3)	−0.0020 (3)	−0.0074 (3)
C17	0.0154 (4)	0.0160 (4)	0.0189 (5)	0.0000 (4)	0.0011 (4)	−0.0034 (4)
C18	0.0132 (4)	0.0132 (4)	0.0192 (4)	−0.0007 (3)	0.0007 (3)	−0.0048 (3)
C19	0.0168 (5)	0.0128 (4)	0.0222 (5)	−0.0005 (4)	−0.0016 (4)	−0.0075 (4)
C20	0.0181 (5)	0.0148 (4)	0.0163 (4)	−0.0009 (4)	−0.0008 (3)	−0.0059 (3)
C21	0.0164 (5)	0.0157 (4)	0.0184 (5)	0.0009 (4)	0.0011 (4)	−0.0051 (4)
C22	0.0207 (5)	0.0180 (5)	0.0267 (5)	−0.0051 (4)	0.0016 (4)	−0.0008 (4)
C23	0.0249 (5)	0.0215 (5)	0.0196 (5)	−0.0082 (4)	−0.0009 (4)	−0.0031 (4)
C24	0.0204 (5)	0.0201 (5)	0.0198 (5)	0.0002 (4)	−0.0045 (4)	−0.0074 (4)
C25	0.0152 (4)	0.0184 (5)	0.0168 (4)	−0.0007 (4)	−0.0028 (3)	−0.0039 (4)
C26	0.0136 (4)	0.0150 (4)	0.0190 (5)	−0.0010 (3)	−0.0023 (3)	−0.0045 (4)
C27	0.0129 (4)	0.0200 (5)	0.0226 (5)	−0.0009 (4)	−0.0022 (4)	−0.0086 (4)
C28	0.0158 (5)	0.0159 (5)	0.0192 (5)	−0.0023 (4)	−0.0022 (4)	−0.0044 (4)
C29	0.0155 (5)	0.0172 (5)	0.0228 (5)	−0.0024 (4)	−0.0007 (4)	−0.0074 (4)
C30	0.0188 (5)	0.0180 (5)	0.0229 (5)	0.0009 (4)	−0.0033 (4)	−0.0019 (4)
C31	0.0253 (5)	0.0213 (5)	0.0216 (5)	0.0008 (4)	−0.0028 (4)	−0.0020 (4)
C32	0.0171 (5)	0.0205 (5)	0.0254 (5)	0.0003 (4)	0.0005 (4)	−0.0068 (4)

### 2,2,5-Trimethyl-1,3-dioxane-5-carboxylic anhydride (II) Geometric parameters (Å, º)

**Table:**

O1—C1	1.3792 (12)	O8—C25	1.3820 (13)
O1—C9	1.4041 (12)	O8—C17	1.4073 (13)
O2—C1	1.1943 (13)	O9—C17	1.1939 (13)
O3—C4	1.4304 (12)	O10—C21	1.4321 (12)
O3—C5	1.4330 (12)	O10—C20	1.4360 (12)
O4—C4	1.4251 (12)	O11—C20	1.4274 (13)
O4—C3	1.4284 (12)	O11—C19	1.4333 (12)
O5—C9	1.1946 (13)	O12—C25	1.1942 (14)
O6—C12	1.4276 (13)	O13—C28	1.4301 (12)
O6—C11	1.4314 (12)	O13—C27	1.4318 (13)
O7—C13	1.4301 (12)	O14—C28	1.4296 (13)
O7—C12	1.4331 (13)	O14—C29	1.4341 (13)
C1—C2	1.5135 (14)	C17—C18	1.5239 (15)
C2—C5	1.5336 (14)	C18—C19	1.5264 (15)
C2—C6	1.5346 (14)	C18—C21	1.5278 (14)
C2—C3	1.5482 (14)	C18—C22	1.5378 (14)
C3—H3A	0.9900	C19—H19A	0.9900
C3—H3B	0.9900	C19—H19B	0.9900
C4—C7	1.5152 (14)	C20—C23	1.5140 (14)
C4—C8	1.5293 (14)	C20—C24	1.5290 (15)
C5—H5A	0.9900	C21—H21A	0.9900
C5—H5B	0.9900	C21—H21B	0.9900
C6—H6A	0.9800	C22—H22A	0.9800
C6—H6B	0.9800	C22—H22B	0.9800
C6—H6C	0.9800	C22—H22C	0.9800
C7—H7A	0.9800	C23—H23A	0.9800
C7—H7B	0.9800	C23—H23B	0.9800
C7—H7C	0.9800	C23—H23C	0.9800
C8—H8A	0.9800	C24—H24A	0.9800
C8—H8B	0.9800	C24—H24B	0.9800
C8—H8C	0.9800	C24—H24C	0.9800
C9—C10	1.5273 (15)	C25—C26	1.5163 (14)
C10—C11	1.5259 (14)	C26—C30	1.5346 (14)
C10—C13	1.5312 (14)	C26—C29	1.5369 (14)
C10—C14	1.5368 (14)	C26—C27	1.5432 (15)
C11—H11A	0.9900	C27—H27A	0.9900
C11—H11B	0.9900	C27—H27B	0.9900
C12—C15	1.5112 (14)	C28—C32	1.5154 (15)
C12—C16	1.5281 (15)	C28—C31	1.5264 (15)
C13—H13A	0.9900	C29—H29A	0.9900
C13—H13B	0.9900	C29—H29B	0.9900
C14—H14A	0.9800	C30—H30A	0.9800
C14—H14B	0.9800	C30—H30B	0.9800
C14—H14C	0.9800	C30—H30C	0.9800
C15—H15A	0.9800	C31—H31A	0.9800
C15—H15B	0.9800	C31—H31B	0.9800
C15—H15C	0.9800	C31—H31C	0.9800
C16—H16A	0.9800	C32—H32A	0.9800
C16—H16B	0.9800	C32—H32B	0.9800
C16—H16C	0.9800	C32—H32C	0.9800
			
C1—O1—C9	118.80 (8)	C25—O8—C17	118.48 (8)
C4—O3—C5	113.91 (7)	C21—O10—C20	114.29 (7)
C4—O4—C3	114.18 (8)	C20—O11—C19	114.15 (8)
C12—O6—C11	113.64 (8)	C28—O13—C27	114.50 (8)
C13—O7—C12	114.10 (8)	C28—O14—C29	114.37 (8)
O2—C1—O1	123.18 (9)	O9—C17—O8	121.02 (9)
O2—C1—C2	126.81 (9)	O9—C17—C18	125.63 (9)
O1—C1—C2	109.90 (8)	O8—C17—C18	113.21 (9)
C1—C2—C5	108.49 (8)	C17—C18—C19	112.87 (8)
C1—C2—C6	111.34 (8)	C17—C18—C21	106.98 (8)
C5—C2—C6	110.85 (8)	C19—C18—C21	106.97 (8)
C1—C2—C3	107.78 (8)	C17—C18—C22	108.84 (9)
C5—C2—C3	107.70 (8)	C19—C18—C22	110.24 (8)
C6—C2—C3	110.56 (9)	C21—C18—C22	110.90 (9)
O4—C3—C2	109.92 (8)	O11—C19—C18	111.17 (8)
O4—C3—H3A	109.7	O11—C19—H19A	109.4
C2—C3—H3A	109.7	C18—C19—H19A	109.4
O4—C3—H3B	109.7	O11—C19—H19B	109.4
C2—C3—H3B	109.7	C18—C19—H19B	109.4
H3A—C3—H3B	108.2	H19A—C19—H19B	108.0
O4—C4—O3	110.43 (8)	O11—C20—O10	110.54 (8)
O4—C4—C7	105.51 (8)	O11—C20—C23	105.06 (9)
O3—C4—C7	105.46 (8)	O10—C20—C23	105.60 (8)
O4—C4—C8	111.28 (8)	O11—C20—C24	111.92 (8)
O3—C4—C8	111.84 (8)	O10—C20—C24	110.91 (8)
C7—C4—C8	112.00 (9)	C23—C20—C24	112.51 (9)
O3—C5—C2	109.66 (8)	O10—C21—C18	109.60 (8)
O3—C5—H5A	109.7	O10—C21—H21A	109.7
C2—C5—H5A	109.7	C18—C21—H21A	109.7
O3—C5—H5B	109.7	O10—C21—H21B	109.7
C2—C5—H5B	109.7	C18—C21—H21B	109.7
H5A—C5—H5B	108.2	H21A—C21—H21B	108.2
C2—C6—H6A	109.5	C18—C22—H22A	109.5
C2—C6—H6B	109.5	C18—C22—H22B	109.5
H6A—C6—H6B	109.5	H22A—C22—H22B	109.5
C2—C6—H6C	109.5	C18—C22—H22C	109.5
H6A—C6—H6C	109.5	H22A—C22—H22C	109.5
H6B—C6—H6C	109.5	H22B—C22—H22C	109.5
C4—C7—H7A	109.5	C20—C23—H23A	109.5
C4—C7—H7B	109.5	C20—C23—H23B	109.5
H7A—C7—H7B	109.5	H23A—C23—H23B	109.5
C4—C7—H7C	109.5	C20—C23—H23C	109.5
H7A—C7—H7C	109.5	H23A—C23—H23C	109.5
H7B—C7—H7C	109.5	H23B—C23—H23C	109.5
C4—C8—H8A	109.5	C20—C24—H24A	109.5
C4—C8—H8B	109.5	C20—C24—H24B	109.5
H8A—C8—H8B	109.5	H24A—C24—H24B	109.5
C4—C8—H8C	109.5	C20—C24—H24C	109.5
H8A—C8—H8C	109.5	H24A—C24—H24C	109.5
H8B—C8—H8C	109.5	H24B—C24—H24C	109.5
O5—C9—O1	120.96 (9)	O12—C25—O8	123.29 (9)
O5—C9—C10	125.75 (9)	O12—C25—C26	126.46 (9)
O1—C9—C10	113.15 (9)	O8—C25—C26	110.18 (9)
C11—C10—C9	113.22 (8)	C25—C26—C30	110.55 (8)
C11—C10—C13	107.29 (8)	C25—C26—C29	108.29 (8)
C9—C10—C13	107.24 (8)	C30—C26—C29	111.02 (9)
C11—C10—C14	109.60 (9)	C25—C26—C27	108.45 (8)
C9—C10—C14	109.05 (8)	C30—C26—C27	111.08 (9)
C13—C10—C14	110.40 (8)	C29—C26—C27	107.34 (8)
O6—C11—C10	111.08 (8)	O13—C27—C26	110.17 (8)
O6—C11—H11A	109.4	O13—C27—H27A	109.6
C10—C11—H11A	109.4	C26—C27—H27A	109.6
O6—C11—H11B	109.4	O13—C27—H27B	109.6
C10—C11—H11B	109.4	C26—C27—H27B	109.6
H11A—C11—H11B	108.0	H27A—C27—H27B	108.1
O6—C12—O7	110.23 (8)	O14—C28—O13	110.38 (8)
O6—C12—C15	105.58 (9)	O14—C28—C32	105.13 (8)
O7—C12—C15	105.40 (8)	O13—C28—C32	105.60 (8)
O6—C12—C16	111.80 (8)	O14—C28—C31	111.89 (9)
O7—C12—C16	111.59 (9)	O13—C28—C31	111.58 (9)
C15—C12—C16	111.91 (9)	C32—C28—C31	111.90 (9)
O7—C13—C10	110.12 (8)	O14—C29—C26	109.88 (8)
O7—C13—H13A	109.6	O14—C29—H29A	109.7
C10—C13—H13A	109.6	C26—C29—H29A	109.7
O7—C13—H13B	109.6	O14—C29—H29B	109.7
C10—C13—H13B	109.6	C26—C29—H29B	109.7
H13A—C13—H13B	108.2	H29A—C29—H29B	108.2
C10—C14—H14A	109.5	C26—C30—H30A	109.5
C10—C14—H14B	109.5	C26—C30—H30B	109.5
H14A—C14—H14B	109.5	H30A—C30—H30B	109.5
C10—C14—H14C	109.5	C26—C30—H30C	109.5
H14A—C14—H14C	109.5	H30A—C30—H30C	109.5
H14B—C14—H14C	109.5	H30B—C30—H30C	109.5
C12—C15—H15A	109.5	C28—C31—H31A	109.5
C12—C15—H15B	109.5	C28—C31—H31B	109.5
H15A—C15—H15B	109.5	H31A—C31—H31B	109.5
C12—C15—H15C	109.5	C28—C31—H31C	109.5
H15A—C15—H15C	109.5	H31A—C31—H31C	109.5
H15B—C15—H15C	109.5	H31B—C31—H31C	109.5
C12—C16—H16A	109.5	C28—C32—H32A	109.5
C12—C16—H16B	109.5	C28—C32—H32B	109.5
H16A—C16—H16B	109.5	H32A—C32—H32B	109.5
C12—C16—H16C	109.5	C28—C32—H32C	109.5
H16A—C16—H16C	109.5	H32A—C32—H32C	109.5
H16B—C16—H16C	109.5	H32B—C32—H32C	109.5
			
C9—O1—C1—O2	3.46 (14)	C25—O8—C17—O9	−55.71 (13)
C9—O1—C1—C2	−179.97 (8)	C25—O8—C17—C18	128.37 (9)
O2—C1—C2—C5	−3.88 (14)	O9—C17—C18—C19	164.90 (10)
O1—C1—C2—C5	179.70 (7)	O8—C17—C18—C19	−19.41 (11)
O2—C1—C2—C6	−126.13 (11)	O9—C17—C18—C21	47.51 (13)
O1—C1—C2—C6	57.46 (10)	O8—C17—C18—C21	−136.79 (8)
O2—C1—C2—C3	112.47 (11)	O9—C17—C18—C22	−72.37 (13)
O1—C1—C2—C3	−63.95 (10)	O8—C17—C18—C22	103.33 (10)
C4—O4—C3—C2	56.70 (10)	C20—O11—C19—C18	−55.78 (11)
C1—C2—C3—O4	−171.01 (8)	C17—C18—C19—O11	−62.23 (11)
C5—C2—C3—O4	−54.14 (10)	C21—C18—C19—O11	55.16 (10)
C6—C2—C3—O4	67.10 (10)	C22—C18—C19—O11	175.83 (8)
C3—O4—C4—O3	−56.64 (10)	C19—O11—C20—O10	53.54 (10)
C3—O4—C4—C7	−170.13 (8)	C19—O11—C20—C23	167.00 (8)
C3—O4—C4—C8	68.19 (11)	C19—O11—C20—C24	−70.62 (11)
C5—O3—C4—O4	57.13 (10)	C21—O10—C20—O11	−55.42 (11)
C5—O3—C4—C7	170.65 (8)	C21—O10—C20—C23	−168.54 (8)
C5—O3—C4—C8	−67.37 (10)	C21—O10—C20—C24	69.31 (10)
C4—O3—C5—C2	−57.84 (10)	C20—O10—C21—C18	58.32 (10)
C1—C2—C5—O3	170.98 (7)	C17—C18—C21—O10	65.26 (10)
C6—C2—C5—O3	−66.48 (10)	C19—C18—C21—O10	−55.93 (10)
C3—C2—C5—O3	54.57 (10)	C22—C18—C21—O10	−176.18 (8)
C1—O1—C9—O5	57.23 (13)	C17—O8—C25—O12	−5.51 (15)
C1—O1—C9—C10	−126.81 (9)	C17—O8—C25—C26	177.25 (8)
O5—C9—C10—C11	−168.32 (10)	O12—C25—C26—C30	121.38 (11)
O1—C9—C10—C11	15.95 (11)	O8—C25—C26—C30	−61.49 (11)
O5—C9—C10—C13	−50.17 (13)	O12—C25—C26—C29	−0.44 (14)
O1—C9—C10—C13	134.10 (8)	O8—C25—C26—C29	176.69 (8)
O5—C9—C10—C14	69.39 (13)	O12—C25—C26—C27	−116.62 (11)
O1—C9—C10—C14	−106.34 (10)	O8—C25—C26—C27	60.51 (10)
C12—O6—C11—C10	56.87 (11)	C28—O13—C27—C26	−56.55 (11)
C9—C10—C11—O6	63.78 (10)	C25—C26—C27—O13	171.20 (8)
C13—C10—C11—O6	−54.34 (10)	C30—C26—C27—O13	−67.12 (10)
C14—C10—C11—O6	−174.23 (8)	C29—C26—C27—O13	54.41 (10)
C11—O6—C12—O7	−55.71 (10)	C29—O14—C28—O13	−55.94 (10)
C11—O6—C12—C15	−169.06 (8)	C29—O14—C28—C32	−169.37 (8)
C11—O6—C12—C16	69.02 (11)	C29—O14—C28—C31	68.96 (11)
C13—O7—C12—O6	56.53 (11)	C27—O13—C28—O14	55.42 (10)
C13—O7—C12—C15	170.01 (8)	C27—O13—C28—C32	168.54 (8)
C13—O7—C12—C16	−68.31 (10)	C27—O13—C28—C31	−69.66 (11)
C12—O7—C13—C10	−57.49 (11)	C28—O14—C29—C26	57.59 (10)
C11—C10—C13—O7	54.30 (10)	C25—C26—C29—O14	−171.68 (8)
C9—C10—C13—O7	−67.63 (10)	C30—C26—C29—O14	66.78 (11)
C14—C10—C13—O7	173.69 (8)	C27—C26—C29—O14	−54.79 (10)

### 2,2,5-Trimethyl-1,3-dioxane-5-carboxylic anhydride (II) Hydrogen-bond geometry (Å, º)

**Table:**

*D*—H···*A*	*D*—H	H···*A*	*D*···*A*	*D*—H···*A*
C3—H3*B*···O10^i^	0.99	2.54	3.5043 (16)	164
C5—H5*A*···O9^ii^	0.99	2.54	3.4723 (18)	156
C11—H11*B*···O10^i^	0.99	2.57	3.5152 (17)	161
C14—H14*A*···O12^iii^	0.98	2.56	3.531 (2)	171
C16—H16*C*···O3^iv^	0.98	2.53	3.4973 (16)	170
C19—H19*A*···O7	0.99	2.53	3.5095 (17)	168
C27—H27*A*···O7	0.99	2.52	3.5035 (16)	170

Symmetry codes: (i) *x*, *y*−1, *z*; (ii) −*x*+1, −*y*+1, −*z*+1; (iii) −*x*+2, −*y*+1, −*z*+1; (iv) *x*+1, *y*, *z*.
