# Supplementary material for: Syntheses and crystal structures of 2,2,5-trimethyl-1,3-dioxane-5-carb­oxy­lic acid and 2,2,5-trimethyl-1,3-dioxane-5-carb­oxy­lic anhydride
Source: Acta Crystallogr E Crystallogr Commun. 2020 Jan 1;76(Pt 1):86–90. doi: 10.1107/S2056989019016670 (PMC6944095; doi:10.1107/S2056989019016670)
Supplement: Supplementary file 4 [file e-76-00086-sup4.docx]

**Supplementary Material**

**For**

**Synthesis and crystal structures of 2,2,5-trimethyl-1,3-dioxane-5-carboxylic acid and 2,2,5-trimethyl-1,3-dioxane-5-carboxylic anhydride**

**by**

Joseph A.  Giesen, Scott M.  Grayson and   Joel T.  Mague*

Department of Chemistry, Tulane University

New Orleans, LA, USA


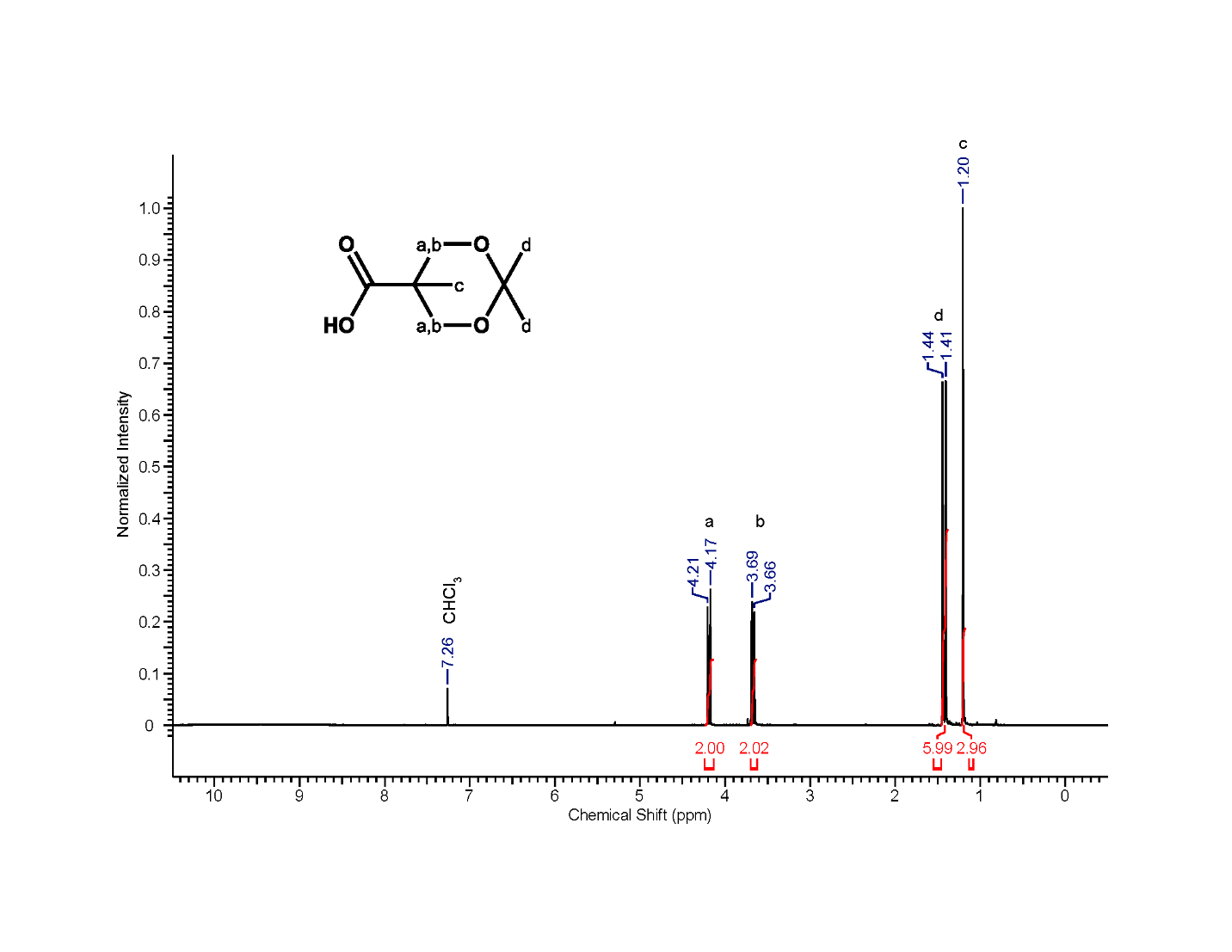


**Figure S1. ^1^H NMR (CDCl_3_, 400 MHz) of I.**

**
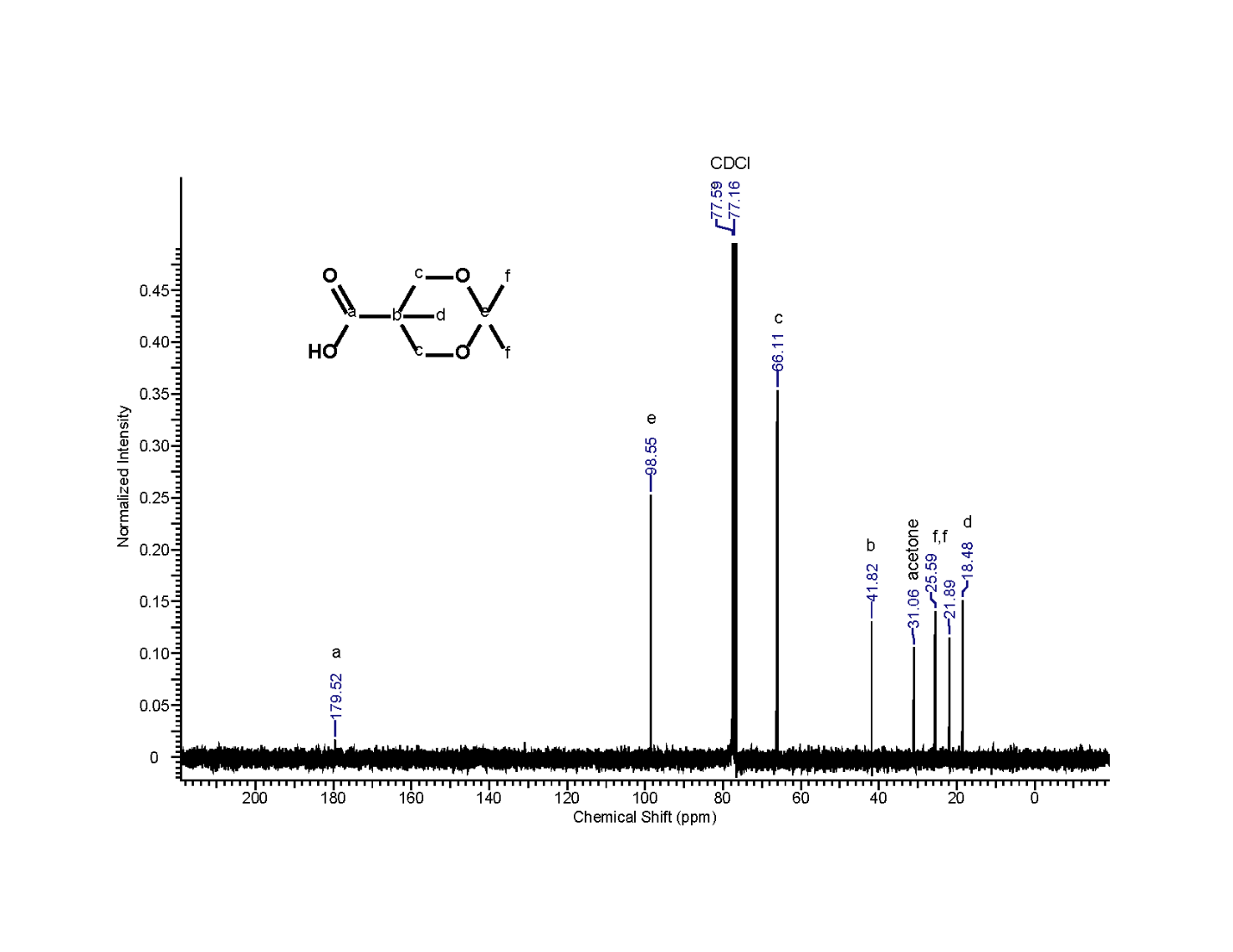
**

**Figure S2. ^13^C NMR (CDCl_3_, 100 MHz) of I.**

**
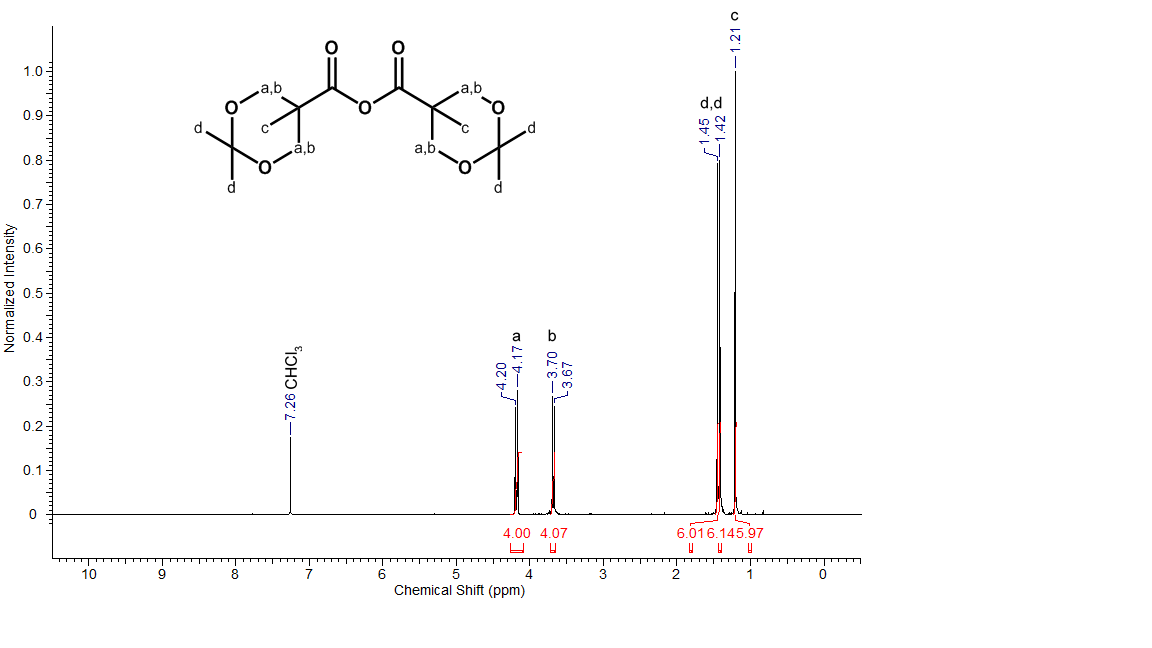
**

**Figure S3. ^1^H NMR (CDCl_3_, 400 MHz) of II.**

**
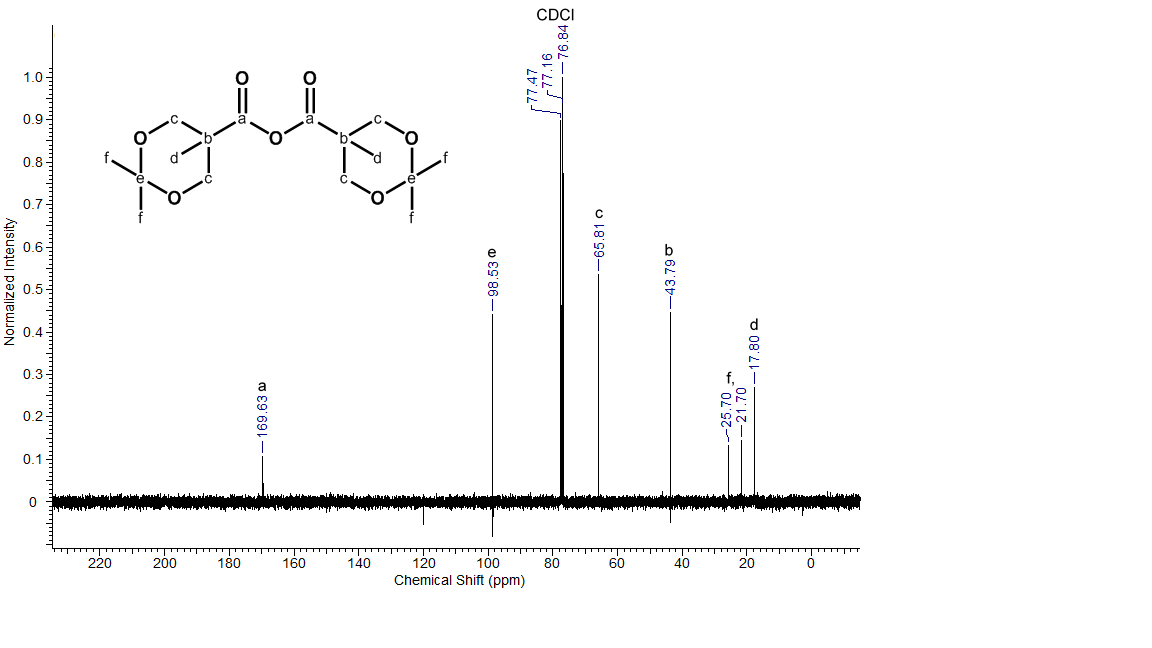
**

**Figure S4. ^13^C NMR (CDCl_3_, 100 MHz) of II.**
